# Regulation of *Nosema ceranae* gene expression by *Bidens pilosa* phytogenic treatment in infected honey bees

**DOI:** 10.3389/finsc.2026.1828903

**Published:** 2026-06-03

**Authors:** Yi-Hsuan Li, Kai-Di Chen, Ju-Chun Chang, Zih-Ting Chang, Chong-Yu Ko, Shang-Tse Ho, Li-Hung Chen, Ming-Cheng Wu, Wen-Chin Yang, Yu-Liang Yang, Yue-Wen Chen, Yu-Shin Nai

**Affiliations:** 1 Doctoral Program in Microbial Genomics, National Chung Hsing University and Academia Sinica, Taichung, Taiwan; 2 Department of Entomology, National Chung Hsing University, Taichung, Taiwan; 3 Department of Entomology, National Taiwan University, Taipei, Taiwan; 4 Department of Biotechnology and Animal Science, National Ilan University, Yilan, Taiwan; 5 Department of Wood-Based Materials and Design, National Chiayi University, Chiayi, Taiwan; 6 Department of Plant Pathology, National Chung Hsing University, Taichung, Taiwan; 7 Advanced Plant Biotechnology Center, National Chung Hsing University, Taichung, Taiwan; 8 Agricultural Biotechnology Research Center, Academia Sinica, Taipei, Taiwan

**Keywords:** *Apis mellifera*, *Bidens pilosa* phytogenic, dsRNA, *Nosema ceranae*, nosemosis, transcriptome

## Abstract

**Introduction:**

*Nosema ceranae* is an obligate microsporidian parasite that causes nosemosis in honey bees (*Apis mellifera*).

**Methods:**

In controlled laboratory cage experiments, newly emerged worker bees were experimentally infected with *N. ceranae spores* and treated with *Bidens pilosa* phytogenic extract (BP). Each treatment group consisted of 50 bees per replicate, and experiments were repeated independently. To explore the molecular basis of BP-associated effects, we conducted transcriptome profiling of *N. ceranae* from infected honey bee midguts at multiple time point. RNA samples from infected bee midguts with BP treatment were collected at 5, 10, and 20 days post-infection (dpi) for transcriptomic analysis.

**Results:**

BP treatment significantly improved survival probability and reduced pathogen load compared to infected controls. During the early infection stage (5 dpi), BP treatment was associated with extensive downregulation of parasite genes, including components of the V-type ATPase pathway. Gene Ontology and KEGG analysis suggested suppression of metabolic and ion transport processes. To further evaluate the potential role of V-type ATPase, RNAi-mediated knockdown resulted in reduced gene expression and showed a trend toward decreased pathogen load and modest improvement in host survival.

**Discussion:**

Although the RNAi results do not provide definitive evidence of causality, they support a potential involvement of the V-type ATPase in parasite proliferation. Overall, BP altered the transcriptomic profile of *N. ceranae* in a stage-dependent manner and may influence parasite development by affecting key metabolic pathways.

## 1 Introduction

The health of honey bees has been a significant concern for both beekeepers and scientists. Multiple factors negatively impact honey bee health, including environmental pollution, food scarcity, poor nutrition, pesticide exposure, and various pathogens (1–4). Among these threats, diseases caused by bacteria, viruses, and fungi are major contributors to colony decline. Notably, nosemosis is highly prevalent in honey bee populations (5–7). This disease is caused by two species of microsporidia, *Nosema apis* and *Nosema ceranae*, which primarily infect adult honey bees (8, 9). Infection with *N. ceranae* weakens honey bees and, in severe cases, can lead to substantial declines in adult bee populations, reduced honey yields, and ultimately, colony collapse. The infection impairs hormone production, lipid synthesis, and midgut tissue integrity (10). It also induces nutritional and energetic stress, suppresses the immune system, and interferes with host cell apoptosis (11). Infected bees exhibit impaired olfactory learning and memory, premature foraging behavior, reduced homing ability, and decreased flight capacity (10, 12). *N. ceranae* infection is associated with increased energetic demand and may contribute to malnutrition and elevated mortality (12, 13).

Given the widespread occurrence of this disease and the limited efficacy of existing treatments, there is a continued need to identify safe and effective control strategies. Researchers have devoted substantial efforts to developing strategies to control *N. ceranae* and mitigate its impact on honey bee populations. While the antibiotic fumagillin was previously considered an effective treatment (14, 15), recent studies have demonstrated its limited efficacy against *N. ceranae* infections (15). Regardless of dosage or method of administration, fumagillin treatment in heavily infected colonies has shown no significant improvement in winter colony size or survival rates (14). Consequently, ongoing research is focused on identifying new, safe, and effective alternatives for the prevention and treatment of *N. ceranae* infections in honey bees. Based on the above-mentioned issue, an alternative approach has been investigated for the management of *N. ceranae* infections. In addition, numerous studies have evaluated the effectiveness of organic extracts and natural supplements, such as commercial products and plant-based supplements (16–18). These include gastric pentadecapeptide BPC 157, BEEWELL AminoPlus, and other commercial products (18, 19). Our previous study also showed that dimethyl sulfoxide (DMSO) has a positive therapeutic effect on honey bees infected with *N. ceranae* (20).

Among these, extracts from a variety of plants have been extensively studied and show potential in controlling *N. ceranae*. Extracts from *Annona squamosa*, *Ocimum basilicum*, *Psidium guajava*, and *Syzygium jambos* have exhibited strong anti-microsporidian activity, inhibiting the development of *N. ceranae* spores, with effect comparable to fumagillin (21). Furthermore, a herbal extract mixture comprising *Rumex acetosella*, *Achillea millefolium*, *Plantago lanceolata*, *Salvia officinalis*, *Thymus vulgaris*, *Rosmarinus officinalis*, and *Laurus nobilis* has demonstrated efficacy in comparison to fumagillin (22).

One such plant, *B. pilosa*, is of particular interest due to its known antiparasitic properties and its role as a food source for bees. *B. pilosa* is an erect, perennial herb belonging to the Asteraceae family. Native to South America, it has spread widely across tropical regions worldwide. In many areas, BP serves as a major pollen source for *A. mellifera*, contributing significantly to bee bread production (23, 24). In Taiwan, *B. pilosa* is commonly used in the production of capsules, decoctions, and tinctures derived from its dried powder, which are marketed as dietary supplements or health food products (25–27). Crude root extracts of *B. pilosa* have demonstrated *in vitro* antiparasitic activity against the protozoan parasite *Plasmodium falciparum* (28, 29). Moreover, phytogenic extracts from *B. pilosa* have shown effectiveness against *Eimeria* parasites, the causative agents of coccidiosis in poultry, while also helping to reduce the risk of drug resistance (27, 30).

As mentioned above, BP has shown inhibitory activity against protistan pathogens; however, limited data are available regarding its effects on nosemosis. Therefore, this study aims to evaluate the effects of BP on *N. ceranae* infection in *A. mellifera*. Based on the transcriptomic analysis, particular attention was given to genes involved in the V-type ATPase pathway. Furthermore, RNA interference (RNAi) was employed to investigate the potential role of this pathway during infection and to further assess BP-associated effects.

## 2 Materials and methods

### 2.1 Honey bees

Honey bees (*A. mellifera*) used in all experiments were collected from colonies maintained at the apiaries of National Ilan University (NIU, Taiwan) and National Chung Hsing University (NCHU, Taiwan). Each selected colony consisted of six to eight frames and was confirmed to have a healthy, laying queen to ensure normal brood development. Colonies were routinely monitored for general health status, and those with low or negligible *N. ceranae* infection levels were selected. Standard beekeeping management practices, including regular syrup feeding and routine *Varroa* mite control, were maintained throughout the study. Brood frames with more than 70-80% of the cells capped were collected and incubated at 34 °C until adult emergence. Newly emerged worker bees were collected and transferred into rearing cages (90 mm in diameter × 55 mm in height). Bees were maintained under laboratory conditions and provided ad libitum access to 50% (w/v) syrup as their food resource.

### 2.2 Extraction of *Bidens pilosa*

BP was extracted with methanol at a 10:1 (v/v, methanol:BP) ratio. Whole parts of *B. pilosa* were collected, washed, air-dried, and ground into powder prior to extraction. The extraction was performed at room temperature, followed by filtration and solvent evaporation under reduced pressure to obtain the crude extract. The extract was stored at −20 °C until use. After extraction, the BP extract was freeze-dried as described previously (30). The dried BP extract was weighed and then dissolved in DMSO to a final concentration of 100 ppm for further use (Sigma Chemical Co., St. Louis, MO).

### 2.3 *N. ceranae* infection and BP treatment

Spores of *N. ceranae* were obtained and purified from the midgut tissues of forager honey bees collected at the apiaries, following protocols described in previous studies (31, 32). The purified spores were quantified using a hemocytometer (Paul Marienfeld GmbH & Co. KG, Baden-Württemberg, Germany). To assess the effects of BP on *N. ceranae* infection, newly emerged adult honey bees were randomly divided into four groups: Blank (honey bees fed with 50% syrup), Nc (honey bees infected with *N. ceranae* and fed with 50% syrup), DMSO+Nc (honey bees infected with *N. ceranae* and fed with 100 ppm DMSO in 50% syrup), and BP+Nc (honey bees infected with *N. ceranae* and fed with 100 ppm BP (dissolved in DMSO) in 50% syrup). For infection, each honey bee was individually fed with 2 μL of *N. ceranae* spore suspension containing 1×10^5^ spores/μL. A total of 50 honey bees were included per group. After inoculation, honey bees were maintained in rearing cages at 34 °C and monitored daily. This experiment was repeated four times for genomic copy number quantification, three times for RNA sequencing (RNA-seq), and four times for quantitative real-time PCR (qPCR) validation. For the RNA-seq, midgut tissues from three honey bees per replicate in each group were collected at 0 (3 hours post-infection, hpi), 5, 10, and 20 days post-infection (dpi) for RNA extraction and library construction.

### 2.4 Quantification of *N. ceranae* genome copy number

Confirmation of *N. ceranae* infection followed the protocol described in our previous study (32). At 0, 1, 3, 5, 10, 15, and 20 dpi, three midguts from each group were collected into 1×TE buffer, homogenized with a pestle, and examined under a light microscope (WHITED, Taiwan). Total DNA was extracted using the EDNA HISPEX tissue kit (Fisher Biotec, Stirling, Australia), then diluted 100-fold. Absolute quantitation of *N. ceranae* genome copy number was conducted via qPCR using primer set: SSU-F/SSU-R (**Supplementary Table 1**) on a CFX Connect Real-Time PCR Detection System (Bio-Rad, California, USA). Each 20 μL reaction contained: 10 μL 2× iQ™ SYBR^®^ Green Supermix, 1 μL of each forward (10 mM) and reverse (10 mM) primer, and 1 μL of 100-fold diluted DNA template. The qPCR reactions were performed using a two-step amplification protocol by the following procedure: initial denaturation at 95 °C for 3 min, 40 cycles of denaturation at 95 °C for 10 s and annealing at 59 °C for 30 s, a final extension at 72 °C for 10 s, and a melting curve at 65 °C to 95 °C with an increase of 0.5 °C in 5 s. All samples were performed in quadruplicate. A standard curve was generated from DNA extracted from a known spore suspension (1×10^7^ spores/100μL), serially diluted 10-fold down to 1×10^0^ spores/mL. Threshold cycle (Ct) values were plotted against the log_10_ of the initial spore concentration, yielding a linear regression equation:: *y* = 3.0383x+42.084 (R^2^ = 0.997; **Supplementary Figure 1**). This approach is consistent with established absolute qPCR quantification techniques using standard curves to derive copy number from Ct values. To correct for the sample preparation, calculated copy numbers were multiplied by the factors: 2 (DNA extraction dilution correction) ×100 (template dilution) ×100 (volume factor, 1 μL of diluted DNA). This experiment was repeated four times.

### 2.5 RNA sequencing and raw data processing

Midgut tissues from honey bees were homogenized using a Tissue Lyser II (QIAGEN, Solingen, Germany). Total RNA was extracted following the manufacturer’s instructions for TRIzol reagent (Life Technologies, USA) in combination with Locking Gel (BIOTOOLS, Taiwan). RNA purity and concentration were assessed using a Nanodrop 1000 Spectrophotometer V3.5 (Thermo Scientific) and a Qubit 2.0 Fluorometer (Life Technologies, USA), respectively. RNA from three biological replicates was pooled for library construction to obtain sufficient RNA quantity and represent an averaged transcriptomic profile (33, 34). While this approach limits statistical inference, therefore, the data was interpreted as exploratory. The mRNA enrichment was performed using poly-A mRNA with oligo-d (T) magnetic beads included in an Ultra RNA Library Prep Kit (NEB, Essex, MA, USA). The mRNA was fragmented, and first- and second-strand cDNA synthesis was conducted using NEBNext random primers followed by end-repair, A-tailing, and adaptor ligation (NEBNext Adaptor). Libraries were enriched via PCR, and quality and quantity were confirmed using an Agilent Fragment Analyzer and qPCR. Sequencing was performed on an Illumina MiSeq platform (301 bp paired-end reads) by Genomics BioSci. & Tech. Co., Ltd. (Taipei, Taiwan). Raw reads were processed by adapter removal using Trimmomatic (35), and quality trimming and filtering were performed with PRINseq (36).

### 2.6 Differentially expressed gene (DEG), GO, and KEGG analysis

Because of *N. ceranae* is an obligate intracellular parasite, it is unculturable, therefore, high-quality reads were aligned to the *N. ceranae* reference genome (ASM98816v1) using HISAT2 with default parameters. Only reads mapped to the *N. ceranae* reference genome were retained for downstream analyses, allowing selective analysis of parasite-associated transcripts while excluding the majority of host-derived sequences. Gene annotation information (GCF_000988165.1_ASM98816v1_genomic.gff) was obtained from the National Biotechnology Information Center (NCBI) (https://www.ncbi.nlm.nih.gov/ accessed on 19 January 2021) for identifying *N. ceranae*-related PE reads. Differential gene expression analysis across experimental groups was performed using Cuffdiff. Genes with zero Fragments Per Kilobase per Million (FPKM) values across all samples were excluded; for the remaining genes, a pseudocount of 1 was added for log transformation (log_2_(FPKM + 1)). Genes exhibiting a log_2_ ratio ≥ 1 or <-1 (fold change ≥ 2 or <- 2) were considered putative differentially expressed between compared groups (DMSO+Nc and BP+Nc) (D/B) for 5, 10, and 20 dpi. Significantly altered genes (DEGs) were subjected to Gene Ontology (GO) enrichment analysis using the hypergeometric test, with a significance defined as *P* < 0.05. For gene orthologue assignment and pathway mapping, they were submitted to the KEGG Automatic Annotation Server (KAAS, http://www.genome.jp/tools/kaas/ accessed on 23 April 2021) (37).

### 2.7 Quantitative PCR (qPCR) validation

To validate the expression patterns of selected DEGs, midgut tissues from three honey bees per group were collected at time points 0, 0.5, 1, 3, 5, 10, 15, and 20 dpi, with four biological replicates per time point. Each sample (~300 μL) was homogenized in Trizol reagent, and total RNA was extracted using the GENEzolTM TriRNA Pure Kit (Geneaid, New Taipei, Taiwan) following the manufacturer’s protocol. RNA purity and concentration were assessed using a Nanodrop 1000 Spectrophotometer V3.5 (EZdrop 1000, New Taipei, Taiwan). cDNA was synthesized from 1 ng/µL of RNA using the BioDiamond RT-PCR mix (Origin Pure BioSci & Tech. Co., Yilan, Taiwan). Quantitative RT-PCR (qRT-PCR) was then performed using gene-specific primer sets (**Supplementary Table 1**). *N. ceranae β-tubulin* was used as an internal control to validate the expression levels of selected DEGs associated with V-type ATPase, including *ATPeV1A* (AAJ76_4700027584), *ATPeV1B* (AAJ76_700039738), *ATPeV1D* (AAJ76_1600056373), *ATPeV1E* (AAJ76_1700024327), *ATPeV0B* (AAJ76_1900038297), *ATPeV0C* (AAJ76_300040219), and *ATPeV0D* (AAJ76_630003653) (**Supplementary Table 1**). The qRT-PCR reactions and thermal cycling were conducted as described above; the annealing temperature of each primer set is summarized in **Supplementary Table 1**. The relative gene expression level was determined by the 2^-ΔCt^ method (log_2_-transformed).

### 2.8 Gene knockdown test

To evaluate the role of *ATPeV0B* in the *N. ceranae* infection, a 212 bp dsRNA (dsVATP), covering nucleotides 253–464 of the gene (**Supplementary Figure 2**), was synthesized. This was achieved via Golden Gate cloning into the pSIG1 vector with the primer sets listed in **Supplementary Table 1** following the protocol (38), and expressed in *E. coli* HT115. The dsVATP was purified with 8 M RNase‐free LiCl (38), air‐dried, and resuspended in 750 µL RNase‐free water for experimental use (39). Four treatment groups (n= 50 honey bees each) were established, including Blank (honey bees fed with 50% syrup), Nc (honey bees infected with *N. ceranae* and fed with 50% syrup), dsGFP+Nc (honey bees infected with *N. ceranae* and fed with GFP dsRNA in 50% syrup), and dsVATP+Nc (honey bees infected with *N. ceranae* and fed with dsVATP in 50% syrup). Infections were performed by feeding newly emerged honey bees 2 μL of a 50% syrup containing 5×10^3^ spores/μL (*N. ceranae*). A low spore concentration was used in the RNAi experiment to avoid excessive infection intensity and allow clearer observation of gene knockdown effects (40, 41). Infections were performed by feeding newly emerged honey bees 2 μL of a 50% syrup containing 5×10^3^ spores/μL (*N. ceranae*). Honey bees were kept at 34 °C with 95% RH in incubators. Starting at 5 dpi, bees in the dsRNA groups received 2 μg dsRNA in syrup every two days. Daily survival was monitored for Kaplan–Meier analysis up to 15 dpi (42). Midgut tissues (three honey bees per group) were collected at 5, 10 (5 days post-dsRNA treatment), and 15 dpi (10 days post-dsRNA treatment) for DNA and RNA extraction as described above. The spore counts, *N. ceranae* genome copies, and expression of *ATPeV1A*, *ATPeV1B*, *ATPeV1D*, *ATPeV1E*, *ATPeV0B*, *ATPeV0C*, and *ATPeV0D* were quantified by qPCR, using the method described above. The relative gene expression level of the dsGFP and dsVATP was determined by the 2^-ΔCt^ method (43, 44). For statistical analysis, values were log_2_-transformed prior to performing Student’s t-test.

### 2.9 Statistical analysis

All statistical analyses and visualization were performed using R version 4.0.3 (45) using various packages, including heatmap, ggplot2, and ggpubr (46–48). Kaplan–Meier survival curves were analyzed using the survminer and survival packages in R, with statistical significance assessed by log-rank (Mantel–Cox) tests and Bonferroni correction for multiple comparisons (42, 49). Spore count and qPCR data, including genome copy and gene expression, were expressed as mean ± standard deviation (SD) from four separate experiments. The differences between groups were analyzed using one-way ANOVA followed by Tukey’s multiple comparisons test. Differences were deemed statistically significant at p<0.05.

## 3 Results

### 3.1 *N. ceranae* infection

**Figure 1:**
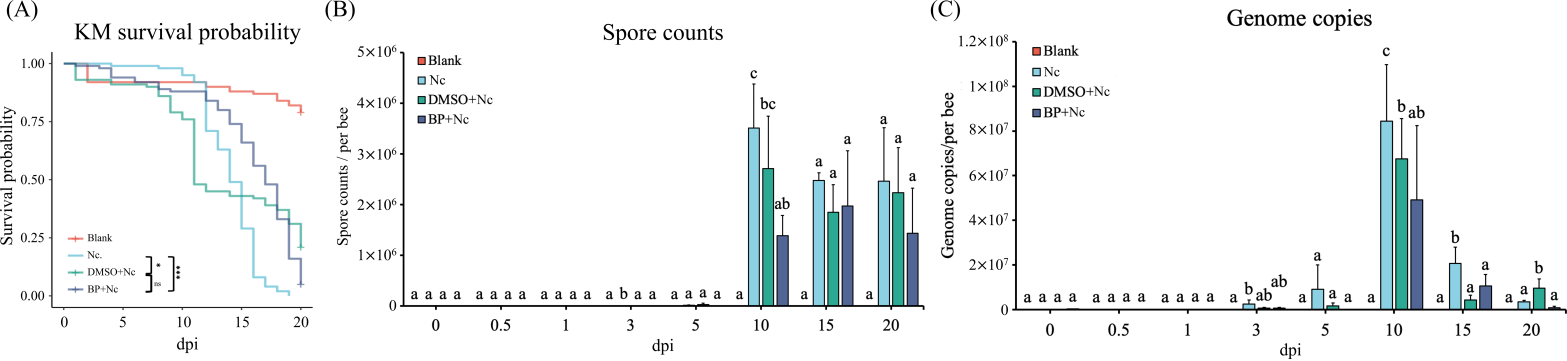
Observation and molecular identification of *N. ceranae* infection. **(A)** Kaplan-Meier survival curves for Blank, Nc, DMSO+Nc and BP+Nc. Pairwise log-rank (Mantel-Cox) tests with Bonferroni correction for multiple comparisons were used to compare each treatment to Nc (*P < 0.05; ***P < 0.001). **(B)** spore counts and **(C)** genome copies. dpi = days post-infection. For **(B, C)**, statistical significance among treatment groups was assessed separately for each time point using one-way ANOVA followed by Tukey’s multiple comparisons test. Data are shown as mean ± SD (n = 4).

Kaplan–Meier survival analysis revealed that the Nc, DMSO+Nc, and BP+Nc exhibited significantly reduced survival compared to the Blank. Among the infected groups, the BP+Nc showed higher survival probability compared to other infected groups, while no significant difference was observed between the BP+Nc and DMSO+Nc (**Figure 1A**). Spores appeared earlier in the Nc (3 dpi) than in the DMSO+Nc and BP+Nc (5 dpi). By 10 dpi, spore counts were significantly highest in the Nc (5.4×10^6^ spores per bee), while the BP+Nc had the lowest count (1.9×10^6^ spores per bee), which was significantly lower than that of the Nc (**Figure 1B**). At later time points, spore counts remained higher in the Nc than in the DMSO+Nc and BP+Nc, although these differences were not statistically significant. Genome copy numbers were detected as early as 0 dpi in all infection groups. Genome copy numbers increased markedly after 10 dpi in all infection groups and then declined thereafter, with relatively lower levels observed in the BP+Nc compared with the Nc (**Figure 1C**).

### 3.2 Sequencing data summary

The relatively low mapping rate reflects the dominance of host-derived RNA in midgut samples. RNA sequencing generated between 1.47 million (M) to 6.85 M across the time points. After quality filtering, the number of clean reads ranged from 1.35 M to 2.09 M. The proportion of reads mapped to the *N. ceranae* genome ranged from 0 to 9.6% in the DMSO+Nc and BP+Nc, while the spore sample showed a higher mapping rate of up to 12% (**Supplementary Table 2**). This low mapping rate likely reflects the predominance of host-derived RNA in whole midgut samples.

### 3.3 DEG analysis

**Figure 2:**
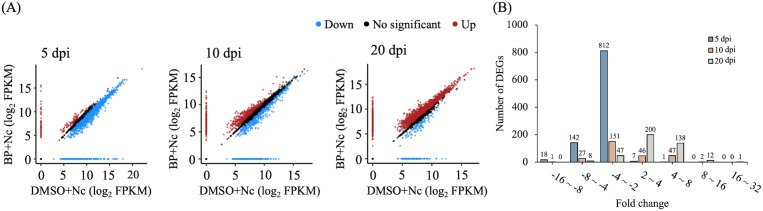
Differential expression analysis between BP+Nc and DMSO+Nc across infection time points. **(A)** Scatter plots of log_2_ FPKM values for BP+Nc versus DMSO+Nc at 5, 10, and 20 days post-infection (dpi). Each dot represents a gene. Genes with |fold change| ≥ 2 are colored: red = significantly upregulated in B/D, blue = significantly downregulated in B/D; non-significant genes are shown in black. **(B)** Histogram showing the number of DEGs in defined fold-change bins at each time point. Fold change of -16 to -8, -8 to -4, -4 to -2 for downregulated; 2 to 4, 4 to 8, 8 to 16, 16 to 32 for upregulated. Numbers above bars indicate DEG counts per bin.

In the D/B comparison, the majority of DEGs were downregulated at 5 dpi, whereas more genes were upregulated at 20 dpi, indicating a time-dependent transcriptional response to BP treatment (**Figure 2A**; **Table 1**). A total of 1006, 431, and 1013 DEGs were identified at 5, 10, and 20 dpi, respectively (**Figure 2B**; **Supplementary Table 3** and **S4**).

**Table 1:** List of the top 10 DEGs by fold change for up- and down-regulated genes at each dpi.

Regulation	dpi	Gene ID	DMSO+Nc FPKM	BP+Nc FPKM	Fold change	NCBI protein (gene) name
Upregulated	5	AAJ76_200015157	60.2937	120.796	4.543928079	dna-directed rna subunit e
		AAJ76_600092331	232.924	467.976	3.922844096	polycomb enhancer epc
		AAJ76_1000131874	144.58	292.413	3.903315287	trna pseudouridine synthase a
		AAJ76_200015201	123.726	250.559	3.787484541	wd40 domain-containing poly + rna export protein
		AAJ76_4000037119	50.4602	102.757	3.378423061	histone acetyltransferase
		AAJ76_4390003	49.2658	102.895	3.270066557	endonuclease-reverse transcriptase, partial
		AAJ76_4500015299	89.9253	192.834	3.03162225	hypothetical protein
		AAJ76_1400060351	100.089	216.495	3.008928865	hypothetical protein
		AAJ76_3500010795	552.637	1200.29	2.98541191	hypothetical protein
		AAJ76_800026843	198.724	443.099	2.977600082	dna replication complex gins protein sld5
	10	AAJ76_2000149458	36.0558	432.635	11.99906428	dna repair protein rad4
		AAJ76_4000150500	31.7441	356.566	11.23252214	iron hydrogenase
		AAJ76_1700061205	4515.31	36076	7.989692616	16S ribosomal RNA
		AAJ76_900082311	44.0685	351.915	7.985650874	mrna capping enzyme
		AAJ76_100094159	901.73	7005.92	7.769436678	aaa atpase domain-containing protein
		AAJ76_2000042761	18.2023	131.245	7.210353541	hypothetical protein
		AAJ76_1780005051	28.8713	206.9	7.16630775	hypothetical protein
		AAJ76_3900033130	13.2096	93.7772	7.09916822	blood-stage membrane protein ag-1
		AAJ76_1000100330	11.1546	78.5205	7.039290378	endoribonuclease dicer
		AAJ76_1300059626	27.746	172.086	6.20219979	hypothetical protein
	20	AAJ76_1000013104	203.338	3317.14	16.31344985	hypothetical protein
		AAJ76_1500043649	41.1108	586.585	14.26838357	20s proteasome subunit
		AAJ76_8000012728	1264.65	2849.97	14.00276669	ring zinc finger domain-containing protein
		AAJ76_730007221	153.752	397.082	13.30097409	pyruvate dehydrogenase e1 component beta subunit
		AAJ76_600045774	17.737	511.393	12.60068274	cullin like protein
		AAJ76_2000125679	77.9985	969.306	12.42720542	skt5-like protein
		AAJ76_700089886	89.3968	182.284	9.790550625	rrp4-like rna-binding protein
		AAJ76_2300055285	271.181	2644.71	9.752553449	dna-directed rna polymerase i
		AAJ76_3300035292	219.438	1977.17	9.01014298	ras-related cell division protein 42-like protein
		AAJ76_280004189	22.8019	191.07	8.379591993	subtilisin-like endopeptidase
Downregulated	5	AAJ76_1600068502	16700.7	1134.28	-14.7236147	ccaat binding transcription factor subunit a
		AAJ76_5000116788	2187.76	160.32	-13.646225	hypothetical protein
		AAJ76_1000166727	1975.56	159.947	-12.3513771	u3 small nucleolar ribonucleoprotein
		AAJ76_1500062863	548.687	45.0673	-12.1748323	hypothetical protein
		AAJ76_2000104532	5865.4	486.924	-12.0458141	d-tyrosyl-trna deacylase
		AAJ76_2200019323	1212.12	102.504	-11.8250961	hypothetical protein
		AAJ76_200084047	1003.13	97.6616	-10.2714646	chromatin assembly factor 1 p60 subunit
		AAJ76_1800016140	2000.5	198.067	-10.1001163	hypothetical protein
		AAJ76_3800025933	2213.85	219.989	-10.0634285	g2 mitotic specific cyclin 1
		AAJ76_100058174	465.535	46.6012	-9.98975987	hypothetical protein
	10	AAJ76_1600024122	493.187	49.1088	-10.0427328	pp-loop atpase
		AAJ76_830001211	364.229	47.3456	-7.69296636	hypothetical protein
		AAJ76_1400063730	3071.49	406.382	-7.55813349	ubiquitin
		AAJ76_300039239	1875.57	265.234	-7.07137123	hypothetical protein
		AAJ76_4200011647	701.319	99.3674	-7.05785602	hypothetical protein
		AAJ76_1000062515	372.986	54.0805	-6.89686152	hypothetical protein
		AAJ76_9000012643	1454.27	212.903	-6.83068375	hypothetical protein
		AAJ76_1400056676	2517.17	456.658	-5.51213033	transmembrane adaptor erv26-like protein
		AAJ76_5600018334	8856.26	1753.21	-5.05145852	protein transport protein yos1
		AAJ76_1300025816	116.532	23.184	-5.02638089	hypothetical protein
	20	AAJ76_1260005835	296.076	50.0031	-5.92116066	chitooligosaccharide deacetylase-like protein
		AAJ76_260003138	143.097	24.7229	-5.78806049	hypothetical protein
		AAJ76_300094826	280.732	48.8358	-5.74847788	tyrosyl-trna synthetase
		AAJ76_860009304	381.28	76.3399	-4.99449911	pol
		AAJ76_930003411	87.4608	18.0246	-4.8523042	hypothetical protein
		AAJ76_900024232	23.9671	5.13648	-4.66604429	dynein heavy chain
		AAJ76_1400045858	761.568	169.459	-4.49412421	transcription initiation factor tfiid subunit
		AAJ76_1600062669	345.306	86.2473	-4.00366149	exosome rnase ph-like protein
		AAJ76_100016514	323.282	80.9325	-3.99445866	hypothetical protein
		AAJ76_1900051261	199.206	50.4844	-3.94588798	hypothetical protein

### 3.4 GO annotation and KEGG pathway analysis

**Figure 3:**
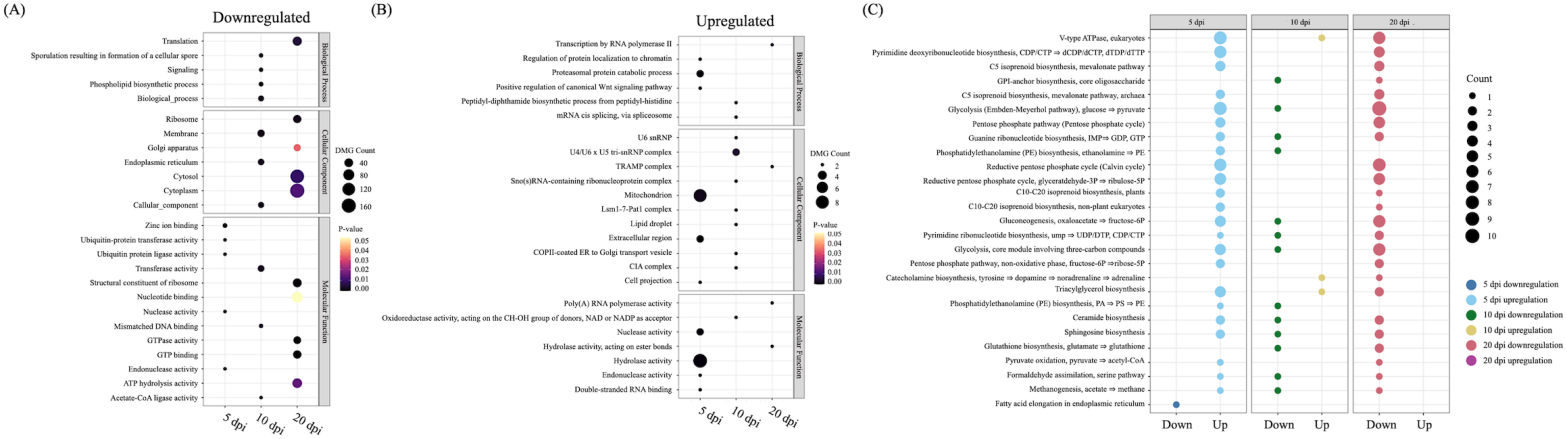
Functional annotation of differentially expressed genes (DEGs) using gene ontology (GO) and KEGG. **(A)** GO annotation of downregulated DEGs; **(B)** GO annotation of upregulated DEGs; **(C)** KEGG pathway annotation of DEGs at 5, 10, and 20 dpi, respectively. GO terms shown are limited to those with the top 10 highest DEG counts and a minimum of 2 associated genes. dpi = days post-infection.

GO annotation analysis revealed time-dependent functional changes in DEGs, indicating dynamic effects of BP treatment on *N. ceranae* gene function (**Table 2****;** **Supplementary Table 5**, **S6**). At 5 dpi, downregulated DEGs were primarily associated with mitochondrion and hydrolase activity, whereas at 20 dpi, upregulated DEGs were enriched in cytosol and cytoplasm (**Figures 3A, B**). These patterns suggest a shift in functional activity during infection progression. The top GO terms for up- and downregulated DEGs at each time point are summarized in **Figure 3A** and **Figures 3B, respectively**, spanning molecular function, cellular component, and biological process categories. KEGG pathway analysis further revealed dynamic changes in metabolic modules and ion-transport processes under BP treatment (**Figures 3C–E**; **Supplementary Table 7**–**S9**). At 5 dpi, genes involved in the V-type ATPase pathway were predominantly downregulated. At 10 dpi, both up- and downregulated pathways were observed, including metabolic modules such as glycolysis and V-type ATPase. By 20 dpi, transcriptional activity increased markedly, with widespread upregulation of metabolic pathways, including V-type ATPase and glycolysis. Overall, genes associated with the V-type ATPase pathway showed downregulation at early stages and upregulation at later stages, suggesting a time-dependent response under BP treatment.

**Table 2:** List of GO terms associated with up- and down-regulated genes, showing the top 10 terms by DEG count and at least two genes per term.

Regulation	dpi	Category	GO term	DMG count	*P-value*
Upregulated	5	Molecular function	zinc ion binding	4	0.001577812
			endonuclease activity	2	0.0000244
			nuclease activity	2	0.000194442
			ubiquitin-protein transferase activity	2	0.000420177
			ubiquitin protein ligase activity	2	0.001262618
	10	Cellular component	membrane	23	0.001634744
			endoplasmic reticulum	14	0.002486499
			cellular_component	11	0.00138483
		Molecular function	zinc ion binding	16	0.002562502
			mismatched DNA binding	3	0.003683504
			acetate-CoA ligase activity	2	0
		Biological process	biological_process	10	0.001590311
			phospholipid biosynthetic process	4	0.0000799
			signaling	3	0.001724481
			sporulation resulting in formation of a cellular spore	3	0.001724481
	20	Cellular component	Cytoplasm	161	0.012354641
			Cytosol	144	0.008864022
			Ribosome	32	0.002205132
			Golgi apparatus	21	0.032790389
		Molecular function	nucleotide binding	80	0.049951026
			ATP hydrolysis activity	54	0.014344335
			structural constituent of ribosome	41	0.000158804
			GTP binding	34	0.00000136
			GTPase activity	26	0.0000157
		Biological process	Translation	46	0.005211188
Downregulated	5	Cellular component	Mitochondrion	8	0.001810113
			extracellular region	3	0.00016989
			cell projection	2	0.001429974
		Molecular function	hydrolase activity	9	0.000602789
			nuclease activity	3	0.000421653
			double-stranded RNA binding	2	0.000838472
			endonuclease activity	2	0.000838472
		Biological process	proteasomal protein catabolic process	3	0.000615712
			regulation of protein localization to chromatin	2	0
			positive regulation of canonical Wnt signaling pathway	2	0.0000453
	10	Cellular component	U4/U6 x U5 tri-snRNP complex	3	0.004526475
			COPII-coated ER to Golgi transport vesicle	2	0.000302933
			CIA complex	2	0.000302933
			Lsm1-7-Pat1 complex	2	0.000302933
			sno(s)RNA-containing ribonucleoprotein complex	2	0.001152103
			lipid droplet	2	0.001152103
			U6 snRNP	2	0.002738914
		Molecular function	oxidoreductase activity, acting on the CH-OH group of donors, NAD or NADP as acceptor	2	0.001152103
		Biological process	mRNA cis splicing, via spliceosome	2	0.001152103
			peptidyl-diphthamide biosynthetic process from peptidyl-histidine	2	0.002738914
	20	Cellular component	TRAMP complex	2	0.00001
		Molecular function	hydrolase activity, acting on ester bonds	2	0.00001
			poly(A) RNA polymerase activity	2	0.0000394
		Biological process	transcription by RNA polymerase II	2	0.004608625

### 3.5 Validation

**Figure 4:**
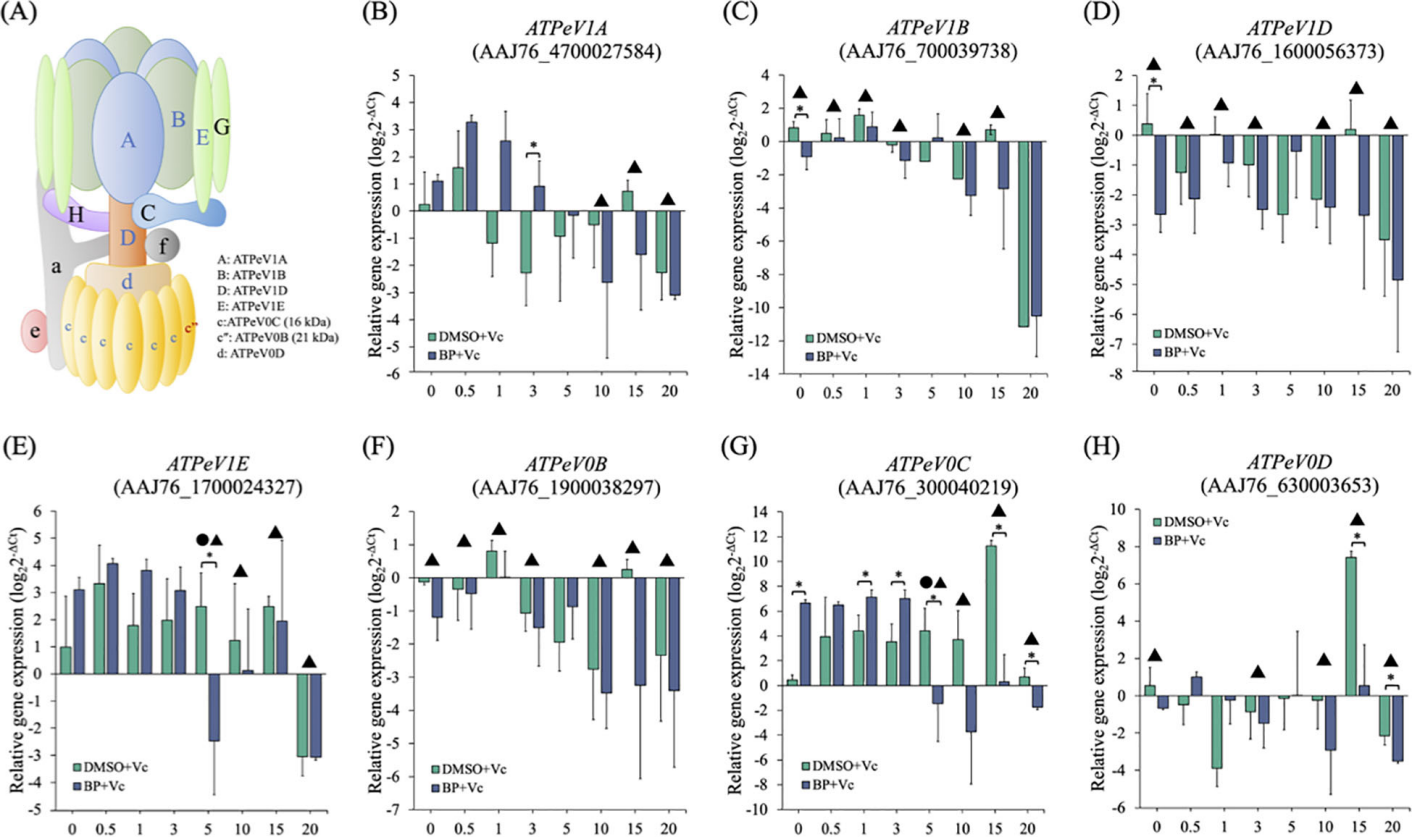
Structural overview of the V-type ATPase and qPCR validation of selected subunit expression. **(A)** Schematic representation of the V-type ATPase complex. The V1 domain (cytosolic side) comprises subunits **(A, B, E, G)**, etc., and the V0 domain (membrane-embedded) comprises subunits a, c, d, etc. Subunits validated in this study are highlighted in blue, and the subunit used for dsRNA treatment is highlighted in red. The structural model was modified from Chen et al., 2022. Relative expression levels of **(B)** *ATPeV1A* (AAJ76_4700027584), **(C)** *ATPeV1B* (AAJ76_700039738), **(D)** *ATPeV1D* (AAJ76_1600056373), **(E)** *ATPeV1E* (AAJ76_1700024327), **(F)** *ATPeV0B* (AAJ76_1900038297), **(G)** *ATPeV0C* (AAJ76_300040219) and **(H)** *ATPeV0D* (AAJ76_630003653) measured by qRT-PCR at the indicated days post-infection (dpi). The results are expressed as log2 relative gene expression (2^-ΔCt^). Relative gene expression levels are presented as mean ± SD (n = 4 biological replicates). At each time point, differences between the DMSO+Nc and BP+Nc were analyzed using an unpaired two-tailed t-test. *P < 0.05. Triangles indicate genes showing lower expression in the BP+Nc compared with the DMSO+Nc, while dots indicate genes showing the same directional trend as observed in the transcriptome data.

To validate the RNA-seq results, the expression of seven V-type ATPase pathway-related genes was examined using qRT-PCR (**Figure 4A**). Among these genes (**Figures 4B–H****),** *ATPeV1A* expression was upregulated at 3 dpi and 10 dpi, followed by downregulation by 20 dpi (**Figure 4B**). For *ATPeV1B*, expression was downregulated at most time points, with the exceptions of 5 and 20 dpi. At 0 dpi, it was significantly downregulated (**Figure 4C**). Similarly, *ATPeV1D* was consistently downregulated at all time points except for 5 dpi and showed significant downregulation at 0 dpi, just like *ATPeV1B* (**Figure 4D**). The expression of *ATPeV1E* was upregulated during the early stages in the BP+Nc, but it began to show significant downregulation at 5 dpi, a trend that aligns with our transcriptome data (**Figure 4E**). *ATPeV0B* showed a similar pattern to *ATPeV1D*, being downregulated at nearly every time point except 5 dpi (**Figure 4F**). *ATPeV0C* was significantly upregulated early on. Consistent with *ATPeV1E*, its expression was downregulated at 5 dpi and matched the transcriptome trends. Following this, *ATPeV0C* showed significant downregulation at the late stage of the experiment (**Figure 4G**). *ATPeV0D* exhibited a distinct pattern from the other V-type ATPase genes, as it was downregulated at 0, 3, 10, 15, and 20 dpi, with significant downregulation observed at 15 and 20 dpi (**Figure 4H**).

*ATPeV0B* displayed the most consistent and pronounced downregulation, suggesting a potential association with the response of *N. ceranae* to BP treatment. Because *ATPeV0B* forms part of the central membrane ring of the V-ATPase complex and exists as a single-copy gene, it was selected for RNAi analysis to further investigate its functional role during infection.

### 3.6 RNAi feeding bioassays

**Figure 5:**
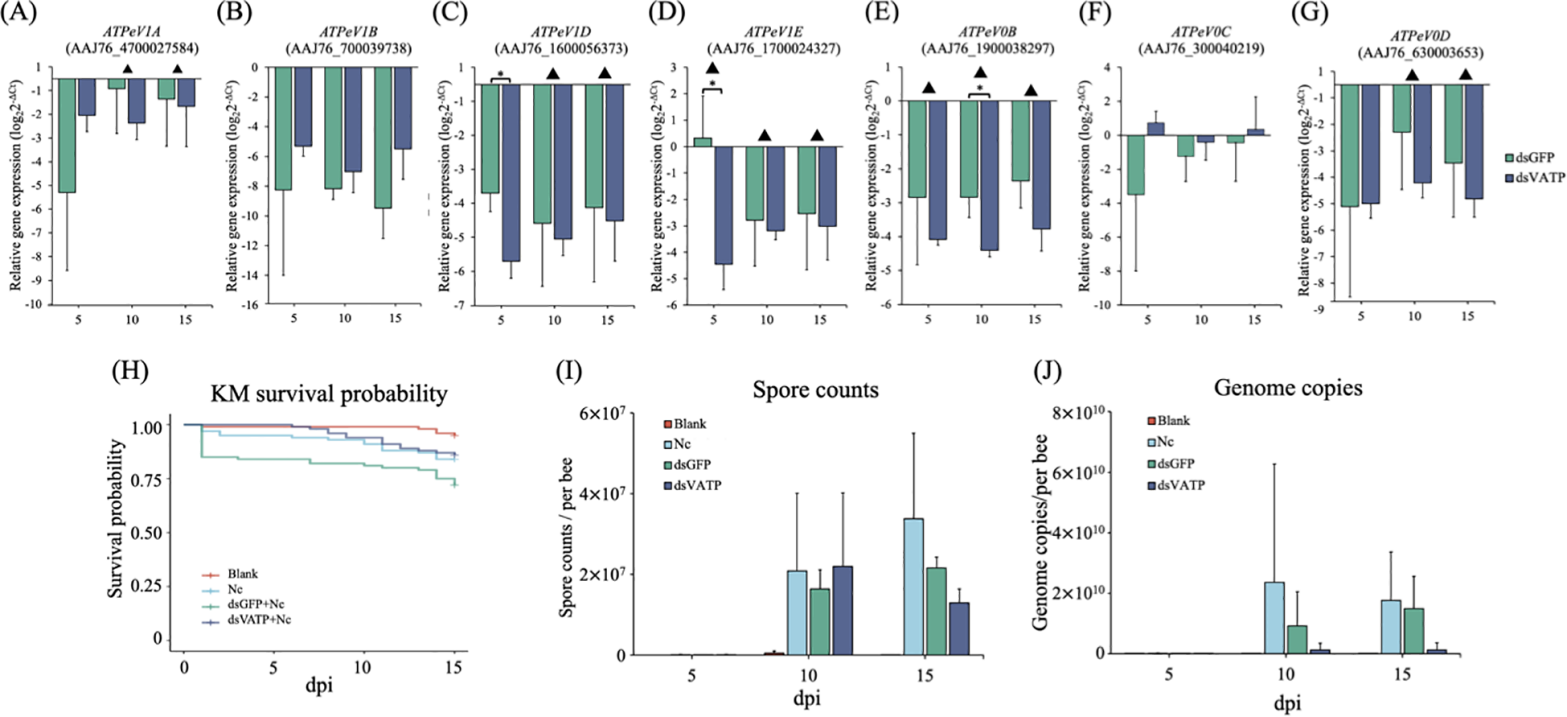
Relative gene expression of V-type ATPase subunit gene and validation in dsVATP-treated samples. **(A–G)** Relative expression of **(A)** *ATPeV1A* (AAJ76_4700027584), **(B)** *ATPeV1B* (AAJ76_700039738), **(C)** *ATPeV1D* (AAJ76_1600056373), **(D)** *ATPeV1E* (AAJ76_1700024327), **(E)** *ATPeV0B* (AAJ76_1900038297), **(F)** *ATPeV0C* (AAJ76_300040219) and **(G)** *ATPeV0D* (AAJ76_630003653) were measured by qRT-PCR at the indicated days post infection (dpi). The results are expressed as log_2_(2^–ΔCt^), and statistical analyses were performed using Student’s *t*-test. Triangles mark genes significantly downregulated by BP compared with the DMSO + Nc. **(H)** Kaplan–Meier survival curves of *N. ceranae* infection in dsGFP+Nc and dsVATP+Nc. Pairwise log-rank (Mantel–Cox) tests with Bonferroni adjustment were used to compare each treatment with Nc. **(I)** spore counts and **(J)** genome copies were analyzed across time points. Statistical significance among time points was assessed by one-way ANOVA followed by Tukey’s multiple comparisons with Bonferroni adjustment. Data are shown as mean ± SD (n = 4).

In the feeding bioassays under a lower infection dose, dsVATP treatment successfully altered the expression patterns of several *V-type ATPase subunits* (**Figures 5A–G**). However, the knockdown effect was not consistently observed at all time points, particularly at 10 and 15 dpi, indicating that the RNAi effect may be transient or limited. As expected, the expression of *ATPeV0B* showed a consistent decrease across time points. Survival analysis showed that the Blank had the highest, whereas all infected groups showed reduced survival (**Figure 5H**). The dsVATP+Nc showed a trend toward higher survival compared with the Nc and dsGFP+Nc, although these differences were not statistically significant. Spore counts and genome copy number increased over time in all infection groups. Even though the dsVATP+Nc showed lower pathogen levels at later time points, these differences were not statistically significant (**Figures 5I, J**). These results suggest that dsVATP treatment may influence the infection dynamics of *N. ceranae*, although the observed effects were modest.

## 4 Discussion

This study showed that treating honey bees with BP provided a noticeable benefit against nosemosis caused by *N. ceranae*. Kaplan-Meier survival analysis showed that BP treatment improved the survival rate of honey bees during infection. Our analysis revealed a discrepancy between spore counts and genome copy numbers at certain time points. While genome copy numbers reflect the total amount of pathogen DNA (including immature or non-viable stages), spore counts measure only the mature, infectious particles. This suggests that BP treatment might have a differential effect on spores and immature or non-viable stages. BP appears to slow down the growth of *N. ceranae*, potentially by influencing gene expression during the early stage of infection. Specifically, BP treatment was associated with changes in genes related to basic metabolic functions, including those in the V-type ATPase pathway. Such changes may affect energy utilization and internal pH regulation, which are important for spore development and replication. *B. pilosa* is recognized as a medicinal plant and a beneficial forage source for honey bees. It contains bioactive compounds such as phenolics and flavonoids (25, 26). Several phenolic compounds have been reported to inhibit *N. ceranae* viability, while flavonoids have also been associated with anti-microsporidian activity in honey bees (50). Additionally, *B. pilosa* has shown anti-coccidial properties in chickens (27). Consistent with these findings, our results indicate that BP not only improves bee survival but also reduces pathogen load. It should also be noted that DMSO, used as the solvent for BP, exhibited a measurable effect on infection dynamics and may partially confound the interpretation of BP-specific effects. Although DMSO exerted a transient effect, BP treatment was associated with a more sustained change in infection dynamics. However, transcriptomic comparisons were performed between the BP+Nc and DMSO+Nc, which helps to partially isolate BP-associated effects, allowing the observed expression differences to be primarily attributed to BP treatment, although the potential influence of DMSO should still be considered.

The time-dependent changes in *N. ceranae* gene expression observed in response to BP treatment suggest a dynamic interaction between the host, pathogen, and compounds. Early in the infection, many genes were downregulated, which may reflect the suppression of key biological processes required for *N. ceranae* infection (12, 32). However, as the infection progressed, this suppression appeared to lessen, and more genes were upregulated. This temporal shift in gene expression suggests that *N. ceranae* may initiate a compensatory or stress-induced response in the later stages. This may indicate that *N. ceranae* activates adaptive mechanisms to mitigate the effects of both BP treatment and RNAi exposure. In fungi such as yeast, stress responses often involve the activation of cellular pathways that help maintain energy homeostasis and cellular integrity under environmental stress (51, 52). Although the stress response mechanisms of microsporidia remain poorly understood, a comparable adaptive response may be triggered in *N. ceranae* to maintain essential cellular functions under treatment-induced stress (53, 54).

This time-dependent pattern implies that BP exerts a stage-specific effect that may inhibit key functions necessary for early infection, while later allowing distinct transcriptional responses as *N. ceranae* attempts to adapt. Similar time-dependent or stage-specific responses have also been reported in other studies (12, 22). These stage-specific dynamics may highlight potential intervention windows, such as targeting the early downregulated pathways to hinder initial establishment, or interfering with later upregulated responses to limit pathogen recovery. Collectively, these findings suggest that BP does not exert a uniform inhibitory effect throughout *N. ceranae* infection, but may modulate its gene expression in distinct and dynamic ways depending on the stage of infection.

GO annotation suggests that BP treatment impacts *N. ceranae* functions in a stage-specific manner. At the early stage of infection (D/B 5 dpi), we observed downregulation of terms such as mitochondrion (GO:0005739) and hydrolase activity (GO:0016787). Although microsporidia lack classical mitochondria, they retain mitosome-derived proteins (55), and hydrolases are involved in nutrient processing and other cellular functions. This pattern suggests that BP may influence energy-related or metabolic processes during early infection, although its direct effect on mitosome components remains unclear. At the late stage (D/B 20 dpi), upregulated GO terms included cytoplasm (GO:0005737) and cytosol (GO:0005829). Typically, cellular formation and replication processes are activated early in microsporidia development. However, their delayed upregulation here may indicate that BP treatment alters the timing of these processes. The cytoplasm in microsporidia contains numerous ribosomes essential for protein synthesis (56), and during sporogony, binary fission leads to sporoblast formation, followed by karyokinesis and cytokinesis (57). The delayed expression of cytoplasm-related genes may therefore be associated with changes in replication dynamics, consistent with the observed delay in infection progression. Altogether, the GO results suggest that BP may influence early-stage metabolic functions and be associated with delay cell division during infection, potentially contributing to slower *N. ceranae* development. The observed downregulation of the V-type ATPase pathway provides a potential link to how BP affects *N. ceranae* physiology. As V-type ATPases are crucial for maintaining intracellular homeostasis and proton transport, changes in their expression may influence conditions required for spore formation and replication. Based on the KEGG analysis, BP treatment was associated with time-dependent changes in metabolic activity. In the early stage of infection, modules involved in energy generation and biosynthesis, such as V-type ATPase (M00160), nucleotide synthesis, glycolysis (M00001), were downregulated. This may reflect reduced ATP-dependent ion transport and metabolic activity during early infection. At 20 dpi, several of these pathways, including the V-type ATPase pathway, were upregulated again, suggesting a possible compensatory response by *N. ceranae* under BP-induced stress. V-type ATPase functions in proton transport across membranes via ATP hydrolysis (58), and may be involved in ion transport and intracellular homeostasis in microsporidia (59). The early downregulation of V-type ATPase-related genes may affect proton transport and ion balance, while the later upregulation may represent an adaptive response to restore cellular equilibrium. Overall, the KEGG results indicate that BP treatment is associated with an initial reduction in metabolic and ion transport activity, followed by a later transcriptional rebound, suggesting a dynamic response of *N. ceranae* to BP treatment across infection stages.

To further evaluate the effect of BP treatment on *N. ceranae* infection and its potential impact on the V-type ATPase pathway, we analyzed the expression levels of seven key V-type ATPase subunits identified as DEGs. This analysis aimed to assess the potential functional relevance of the V-type ATPase pathway in the context of infection and treatment. The timing of this expression peaks roughly coincided with increases in spore counts and genome copy numbers, suggesting that V-type ATPase activity may be associated with *N. ceranae* development. Although a causal relationship remains to be confirmed, the observed expression patterns are consistent with this possibility, particularly given the established role of V-type ATPases in energy metabolism, ion homeostasis, and intracellular trafficking in other eukaryotic systems (60, 61). V-type ATPases are multi-subunit pumps involved in intracellular acidification and various cellular processes. Some subunits contribute to structural stability, while others form the proton-conducting channel. For example, the V0 proteolipid ring has been shown to function as a membrane-spanning pore (62). Based on its central role in proton transport and its consistent expression pattern, *ATPeV0B* was selected for RNAi-mediated knockdown to explore its potential function during infection. Our qPCR results showed that *ATPeV0B* expression was consistently lower under BP treatment. Although *ATPeV1D* exhibited a similar tread, *ATPeV0B* was prioritized due to its role in the V0 domain, which is directly involved in proton transport, whereas *ATPeV1D* primarily contributes to the catalytic V1 domain (60). The infection dose used in the RNAi experiment (5×10³ spores per bee) was intentionally lower than that used in the BP efficacy experiment (1×10^5^ spores per bee) to avoid excessive mortality and allow longitudinal observation of dsRNA treatment effects. While RNAi-mediated knockdown of ATPeV0B was associated with a partial reduction in *N. ceranae* load and a modest improvement in host survival, these differences were not statistically significant. Therefore, although the results suggest a possible involvement of *ATPeV0B* in infection dynamics, they do not provide definitive evidence of its functional role. Instead, these findings support the idea that the V-type ATPase pathway may be relevant to parasite development and warrants further investigation.

While the dsRNA treatment specifically targeted *ATPeV0B*, several V-type ATPase-related genes showed expression changes similar to those observed under BP treatment. This suggests that BP may affect multiple pathways, including the V-type ATPase pathway, in *N. ceranae*. The upregulation of the *ATPeV0C* subunit, a major component of the c-ring that functions together with *ATPeV0B* (62), may represent a compensatory response to changes in *ATPeV0B* expression. This observation suggests that *N. ceranae* may exhibit a degree of transcriptional flexibility under treatment conditions.

Although RNAi treatment was designed to target *ATPeV0B*, the observed effects on gene expression and pathogen load were modest and not statistically significant. Therefore, while changes in related subunits were observed, these patterns should be interpreted with caution, and do not provide clear evidence of a compensatory mechanism. Instead, they may reflect broader transcriptional responses to treatment. Despite these limitations, the altered expression of multiple V-type ATPase-related genes suggests that perturbation of this pathway may influence overall cellular function. Although dsVATP treatment was associated with a reduction in pathogen load, the differences were not statistically significant, which may be due to factors such as insufficient dsRNA dosage or transient knockdown effects. These findings suggest that targeting a single gene or pathway may not be sufficient to achieve a strong therapeutic effect against a complex pathogen. In contrast, BP treatment, which likely affects multiple pathways simultaneously, may exert broader effects on infection dynamics.

## Data Availability

The datasets presented in this study can be found in online repositories. The names of the repository/repositories and accession number(s) can be found in the article/**Supplementary Material**.

## Supplementary material

Supplementary Figure 1 The standard curve of genome copies.

Supplementary Figure 2 The position of dsVATP on *ATPeV0B*.

Supplementary Table 1 Primer list.

Supplementary Table 2 RNA sequencing summary.

Supplementary Table 3 Up-regulated DEG list of D/B comparison group.

Supplementary Table 4 Down-regulated DEG list of D/B comparison group.

Supplementary Table 5 Up-regulated GO list of D/B comparison group.

Supplementary Table 6 Down-regulated GO list of D/B comparison group.

Supplementary Table 7 Up-regulated KEGG list of D/B comparison group.

Supplementary Table 8 Down-regulated DEG list of D/B 20 dpi comparison group.

Supplementary Table 9 KEGG module number of the D/B comparison group.
