# Supplementary figures and images for: Regulation of Nosema ceranae gene expression by Bidens pilosa phytogenic treatment in infected honey bees
Source: Front Insect Sci. 2026 Jun 3;6:1828903. doi: 10.3389/finsc.2026.1828903 (PMC13273367; doi:10.3389/finsc.2026.1828903)

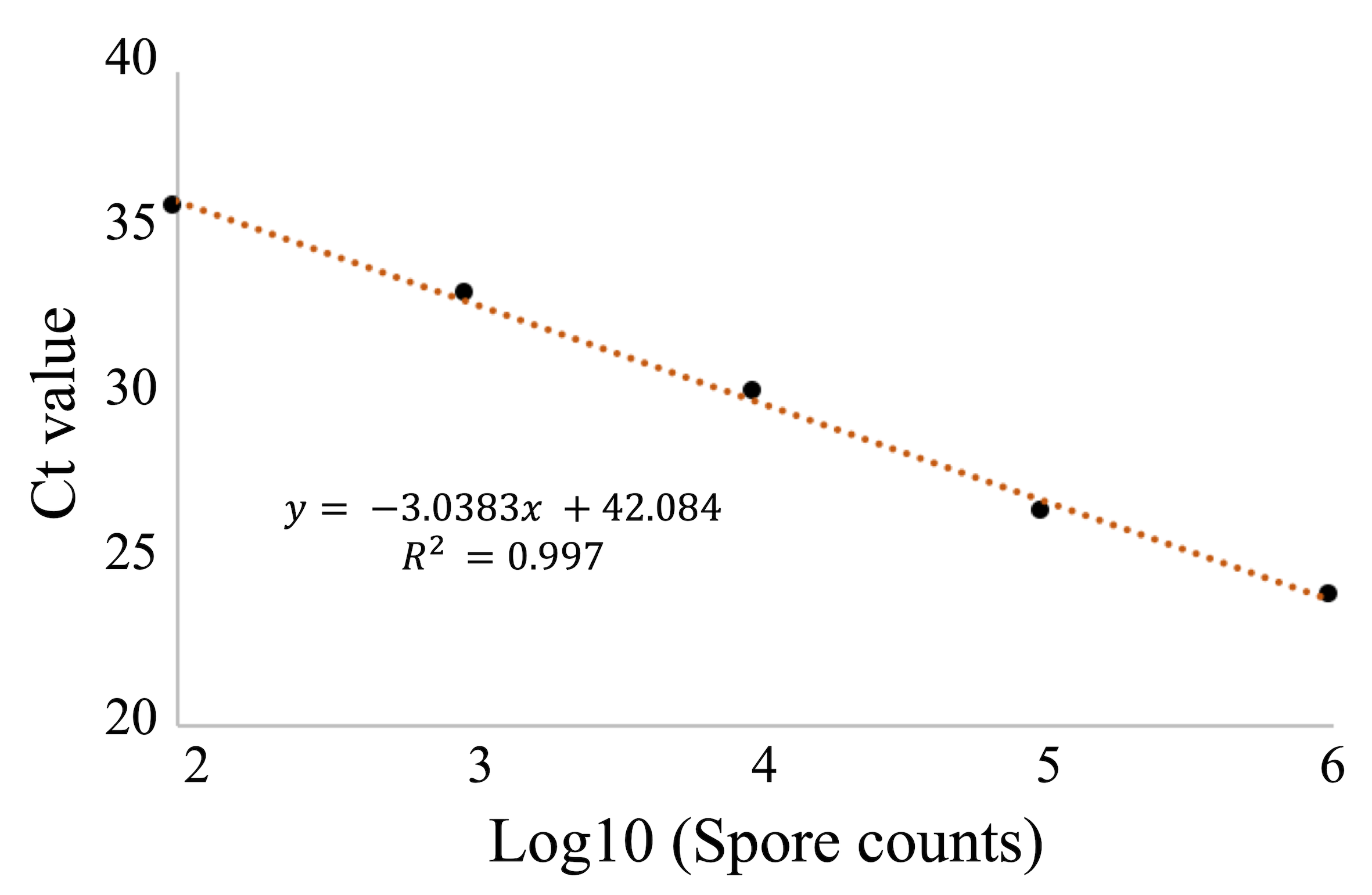

Supplement: Supplementary Figure 1 — The standard curve of genome copies. [file Image1.tiff]

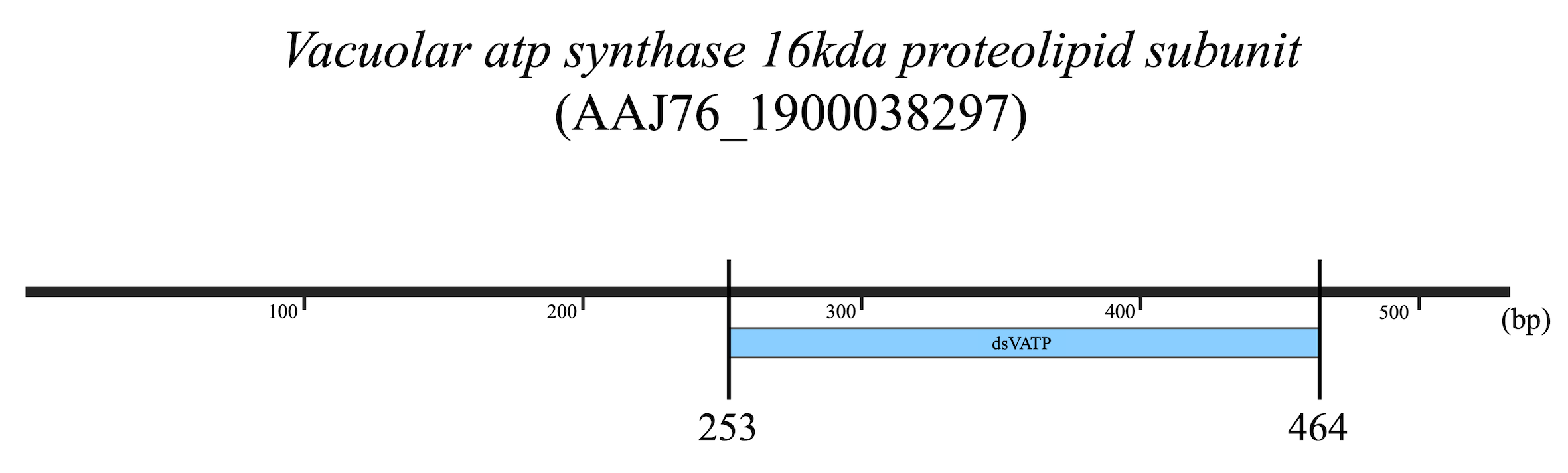

Supplement: Supplementary Figure 2 — The position of dsVATP on ATPeV0B. [file Image2.tiff]
